# Chromite chemistry of a massive chromitite seam in the northern limb of the Bushveld Igneous Complex, South Africa: correlation with the UG-2 in the eastern and western limbs and evidence of variable assimilation of footwall rocks

**DOI:** 10.1007/s00126-020-00964-y

**Published:** 2020-03-03

**Authors:** Malose M. Langa, Pedro J. Jugo, Matthew I. Leybourne, Danie F. Grobler, Jacob Adetunji, Henrik Skogby

**Affiliations:** 1 Mineral Exploration Research Centre, Harquail School of Earth Sciences, Laurentian University, Sudbury, Canada; 2 Department of Geological Sciences and Geological Engineering, Queen’s University, Kingston, Ontario Canada; 3 McDonald Institute, Queen’s University, Kingston, Ontario Canada; 4 Ivanplats (Pty) Ltd., Mokopane, South Africa; 5 Department of Natural Sciences, College of Life and Natural Sciences, University of Derby, Derby, UK; 6 Swedish Museum of Natural History, Department of Geosciences, Stockholm, Sweden

**Keywords:** Bushveld, Chromite, Platreef, UG-2, Trapped liquid shift effect

## Abstract

The Bushveld Igneous Complex (BIC) is known for its laterally extensive platinum group element–bearing layers, the most famous being the Merensky Reef and the UG-2 chromitite in the eastern and western limbs of the complex. In the northern limb, the Platreef mineralization and a thick chromitite seam below it (referred to as the “UG-2 equivalent” or UG-2E) have been proposed to be the stratigraphic equivalents of the Merensky Reef and the UG-2, respectively. In this study, we compare a suite of UG-2E samples from the Turfspruit project with a UG-2 reference suite from the western limb using petrography, electron probe microanalysis, laser ablation-inductively coupled plasma-mass spectrometry, and Mössbauer spectroscopy. The results show that (a) in Mg# vs. Cr# diagrams, UG-2E chromites have a distinct compositional field; however, when samples of similar chromite modal abundance (≥ 80%) are used, the UG-2E chromites overlap the field that characterizes UG-2 chromites; (b) the UG-2E is more variable in chromite modal abundance than the UG-2; and (c) variations in Mg# and Fe^3+^/ΣFe in the UG-2E indicate contamination of the magma by metasedimentary rocks of the Duitschland Formation (Transvaal Supergroup) during emplacement, followed by partial re-equilibration of chromite grains with a trapped melt. Thus, we conclude that for chromite modes higher than 80%, the chromite composition retains enough information to allow correlation and that the UG-2E in the northern limb is very likely the UG-2 chromitite.

**Electronic supplementary material:**

The online version of this article (10.1007/s00126-020-00964-y) contains supplementary material, which is available to authorized users.

## Introduction

The Upper Critical Zone (UCZ) of the Rustenburg Layered Suite (RLS) of the Bushveld Igneous Complex (BIC) contains the largest platinum group element (PGE) reserves in the world (USGS 2019). The RLS consists of ultramafic to mafic cumulate rocks primarily exposed at three limbs: eastern, western, and northern. These limbs host PGE mineralization in layers referred to as “reefs,” namely, UG-2, Merensky Reef, and Platreef. The western and eastern limbs host PGE ores within the UG-2 and the Merensky Reef, whereas ores within the northern limb are associated with the Platreef. Although there is a general consensus that the Platreef is stratigraphically the lateral equivalent of the UCZ in the rest of the BIC, there is no consensus on whether the Platreef mineralization correlates with the Merensky Reef or constitutes a different rock unit formed from a different magma (Wagner 1929; White 1994; Manyeruke et al. 2005; McDonald et al. 2005; McDonald and Holwell 2007; Maier et al. 2008; Kinnaird and McDonald 2005; Yudovskaya and Kinnaird 2010; Grobler and Nielsen 2012; Yudovskaya et al. 2017a, b; Grobler et al. 2019). Deep drilling by Ivanplats on their Turfspruit project intersected a massive chromitite seam below high-grade mineralization. This high-grade mineralization in the upper part of the Platreef has been interpreted to be the equivalent of the Merensky Reef, and the underlying chromitite has been interpreted as the equivalent of the UG-2 seam that is present in the eastern and western limbs of the BIC (Grobler et al. 2019). Chromite is of interest for several reasons. First, it is an early cumulate mineral (Roeder and Reynolds 1991). Second, it is a key mineral in all the PGE-mineralized reefs (McLaren and De Villiers 1982; Cawthorn et al. 2002; Yudovskaya and Kinnaird 2010). Third, it has relatively high closure temperatures (> 800 °C; Van Orman and Crispin 2010); therefore, its mineral chemistry likely preserves information about the conditions of crystallization in its crystal chemistry (Irvine 1965, 1966; Dick and Bullen 1984; Voigt and von der Handt 2011; Junge et al. 2014). In this study, we used electron probe microanalysis (EPMA), laser ablation-inductively coupled plasma-mass spectrometry (LA-ICP-MS), and Mössbauer spectroscopy to investigate the compositional variability of chromite from the UG-2 equivalent in the northern limb (henceforth labeled as UG-2E) compared with that of chromite from the UG-2 to infer possible correlations between the two and to assess how footwall assimilation and the trapped liquid shift effect (Barnes 1986) may have affected the composition of the UG-2E chromites.

## Regional geology of the Bushveld Igneous Complex

The 2054.4 ± 3-Ma Bushveld Igneous Complex (Scoates and Friedman 2008) in South Africa is the largest known igneous intrusion in the world. It stretches approximately 350 km north to south and 450 km east to west and has an estimated total thickness of 9 km (Vermaak 1976; Eales and Cawthorn 1996). The BIC was intruded within the central portions of the Kaapvaal Craton into Transvaal Supergroup metasedimentary rocks as well as Archean granite-gneiss basement rocks in the northern part of the northern limb (Cawthorn et al. 1985; Zeh et al. 2015). The BIC contains the world’s largest known mafic-ultramafic layered complex, the Rustenburg Layered Suite (Eales and Cawthorn 1996). The Rustenburg Layered Suite consists, from base to top, of the following: the Marginal Zone, the Lower Zone, the Critical Zone, the Main Zone, and the Upper Zone, as described elsewhere (Eales and Cawthorn 1996; Maier et al. 2013; Viljoen 2016). The following sections emphasize only aspects pertinent to chromitite seams.

### PGE reefs

The UG-2 is a PGE-mineralized massive chromitite layer with an average thickness of approximately 1 m but varies between 0.4 and 2.5 m, depending on locality (Junge et al. 2014). The Merensky Reef has been described as a PGE-mineralized, chromite-bearing package of predominantly pyroxenitic rocks with thickness ranging from several centimeters up to 20 m, but with an average of 1 m (Cawthorn et al. 2002; Latypov et al. 2015). The PGE mineralization of the Merensky Reef is almost always associated with thin chromitite stringers (Cawthorn et al. 2002). In contrast to the UG-2, which has well-defined petrological boundaries, the term Merensky Reef is mostly a mining term (Vermaak 1976; Cawthorn et al. 2002). The typically quoted average thickness of 1 m for the Merensky Reef is not defined by lithological changes, but by the grade distribution of PGE, usually around thin chromitite seams (Cawthorn et al. 2002). In the northern limb, PGE mineralization is hosted within the Platreef. However, in contrast to the UG-2 and the Merensky Reef, the term Platreef is not restricted to the relatively narrow units containing PGE mineralization but it has been used also to describe all the lithologies between the Main Zone and the footwall metasedimentary rocks or gneisses. Thus, the Platreef is loosely defined as a variably PGE-mineralized package of mafic-ultramafic rocks, primarily pyroxenites, of variable thickness that contains chromitite layers, has a thickness that varies from less than 50 m and up to approximately 600 m, and contains metasedimentary xenoliths of the floor rocks (Van der Merwe 1976; Gain and Mostert 1982; Manyeruke et al. 2005; Maier et al. 2008; Yudovskaya and Kinnaird 2010; Kekana 2014; Yudovskaya et al. 2014).

Grobler et al. (2019) described the Platreef on the Turfspruit project as a suite of mafic-ultramafic rocks beneath the Main Zone, containing a Merensky Reef equivalent with an average thickness of 20 m, which is underlain by a UG-2 analogue chromitite seam, as well as other unmineralized mafic-ultramafic rocks.

### Bushveld chromitites and the UG-2

Bushveld chromitite seams of the Rustenburg Layered Suite within the eastern and western limbs of the BIC are one of the defining characteristics of the Critical Zone. The chromitite seams are separated into three groups: Lower Group (LG), Middle Group (MG), and Upper Group (UG) chromitites, from bottom to top (Cousins and Feringa 1964; Gain 1985). Within each group, the chromitite seams are labeled sequentially from top to bottom: LG-1 to LG-7, MG-1 to MG-4 (overlapping the boundary between the Lower Critical Zone and the UCZ), and UG-1 to UG-3, although the UG-3 is only present within some sections of the eastern limb (Gain 1985; von Gruenewaldt et al. 1986). Thus, with few exceptions, the UG-2 chromitite is the uppermost major chromitite seam in the BIC and the major chromite seam below the Merensky Reef. It is variably separated from the Merensky Reef by a distance of 10 to 400 m (McLaren and De Villiers 1982). Typically, the UG-2 chromitite seam is a massive chromitite, consisting of 60–90 vol.% chromite with an average Cr/Fe value between 1.3 and 1.4, and an average of 43.5 wt% Cr_2_O_3_ (Eales and Reynolds 1986; Lee 1996; Mathez and Mey 2005; Naldrett et al. 2009; Junge et al. 2014). The footwall of the UG-2 is typically a feldspathic pyroxenite (which can be pegmatoidal), anorthosite, or norite, and the hanging wall is usually pyroxenitic (Scoon and de Klerk 1987; Mondal and Mathez 2006). Mining of the PGE from chromitite is generally limited to the UG-2 seam, which contains up to 10 ppm Σ [PGE + Au], with PGE grades typically around 4–8 g/t and Pt/Pd values close to unity, with relatively low content of base metal sulfides (Maier and Barnes 2008; Junge et al. 2014; Veksler et al. 2015). Studies of chromite composition (Cousins and Feringa 1964; Scoon and Teigler 1994; Naldrett et al. 2009, 2012) show systematic differences between the LG, MG, and UG chromitites, suggesting that chromite chemistry may be sufficiently distinct to assist in their correlation.

### Origin of chromitite seams

The processes leading to the formation of chromitite layers are still debated. Their origin is thought to involve as follows: continued magma injections and chromite saturation as a result of magma mixing (Irvine 1975; Sharpe and Irvine 1983); changes in pressure which influence chromite stability (Cameron 1977; Lipin 1993; Latypov et al. 2018); changes in *f*O_2_ (Ulmer 1969); contamination of a new magma pulse by acidic (silica-rich) material inside the chamber (Irvine 1975; Alapieti et al. 1989; Kinnaird et al. 2002); injection of new pulses of magma carrying chromite in suspension (Eales 2000; Mondal and Mathez 2006; Maier and Barnes 2008; Eales and Costin 2012); volatile fluxing (Nicholson and Mathez 1991).

### The northern limb, the Platreef, and the Flatreef

The northern limb of the BIC is separated from the rest of the BIC by the Thabazimbi-Murchison Lineament (TML), an inferred crustal-scale structure (Zeh et al. 2015; Grobler et al. 2019). From the Zebediela fault, the northern limb extends to the north for approximately 110 km until it dips below the Waterberg sedimentary package (Yudovskaya and Kinnaird 2010). Although the northern limb consists of rocks that are locally different from the other limbs, a series of intrusive rocks identified as the Upper Zone, the Main Zone, and the Lower Zone are present, together with the Platreef (Jones 2013; Grobler et al. 2019). The Platreef is found immediately below rocks recognized as part of the Main Zone, hence at the same stratigraphic position as the UCZ in the western and eastern limbs (Kinnaird et al. 2005; Yudovskaya et al. 2017a, b). North of Mokopane, on the Turfspruit project, the ~ 40° westerly dipping unit flattens out for about 1–2 km and PGE-Ni-Cu mineralization becomes subhorizontal at a depth of ~ 600 m in what is known as the Flatreef (Kekana 2014; Grobler et al. 2019). The steeper dip resumes towards the western portion of the project. Also, unlike other areas where the Platreef has been studied near surface or on exposed lithologies, the Flatreef preserves well-mineralized, thickened magmatic rock layers, especially towards the west of the project (Yudovskaya et al. 2017a, b; Grobler et al. 2019); a relatively uncontaminated Platreef sequence has also been recognized and described at Akanani, north-west of Turfspruit (Mitchell and Scoon 2012). In essence, towards the west and at depth, the complex intermingling of magmatic and sedimentary rocks becomes less pronounced and the Platreef gradually resembles the UCZ as recognized in the main limbs of the BIC. This led to the use of descriptive nomenclature equivalent to those used for the UCZ in the rest of the BIC, including a Merensky Cyclic Unit and a UG-2 Cyclic Unit (Grobler et al. 2019). In addition, it has been observed that chromitite seams become more prevalent with less contamination downdip in the Flatreef (Yudovskaya et al. 2017a). Details of the stratigraphy of the Flatreef are summarized in Grobler et al. (2019). This contribution focuses on the UG-2 Cyclic Unit, which consists of three rock units: the UG-2 hanging wall (UG2HW; mainly pyroxenite), the massive to semi-massive chromitite UG-2 analogue (the UG-2E), and the UG-2 footwall (UG2FW; pegmatoidal pyroxenite and harzburgite). Correlation with the UG-2 is mostly based on thickness (roughly 1 m) and stratigraphic position (first massive chromitite below the MCU). However, the UG-2E shows variable interaction with the sedimentary footwall and has lower PGE contents (2 to 6 ppm) than the UG-2 in the eastern and western limbs.

## Samples and analytical methods

**Fig. 1:**
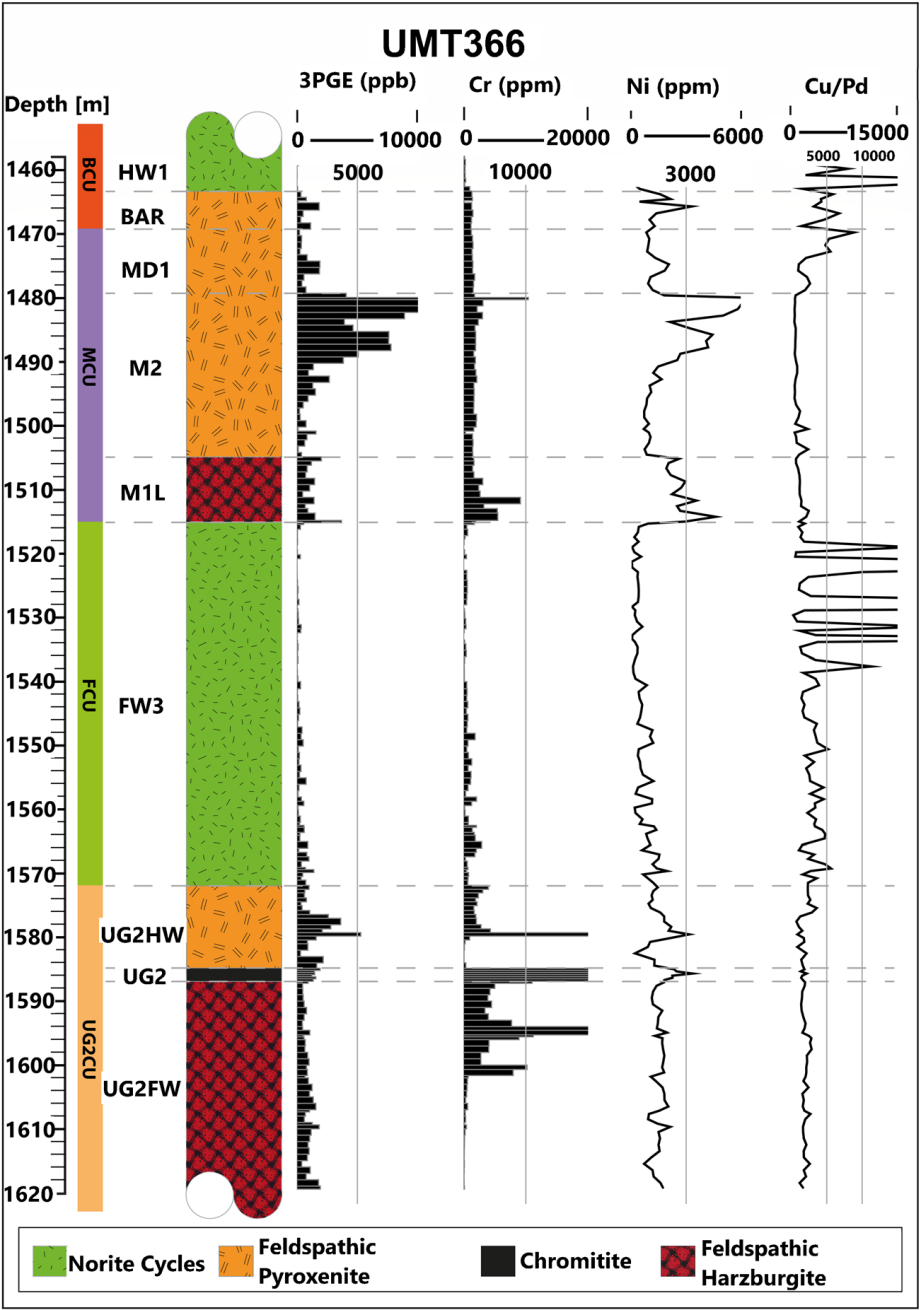
Stratigraphy of drill-hole UMT366 as well as 3PGE (Pd + Pt + Au), Cr, Ni, and Cu/Pd contents in whole rock. Stratigraphic nomenclature from Grobler et al. (2019). The stratigraphy here is characteristic of the Platreef stratigraphy at Turfspruit. The BCU (Bastard Cyclic Unit) consists of the HW1 (hanging wall 1: norite cycles) and the BAR (Bastard Reef: mineralized pyroxenitic unit); the MCU (Merensky Cyclic Unit) consists of the MD1 (Middling Unit 1: weakly mineralized feldspathic pyroxenite), M2 (Merensky Reef analogue: mineralized feldspathic pyroxenite bounded by mm-thick chromitite stringers), and the M1L (M1 Lower: mineralized pegmatoidal feldspathic harzburgite); the FCU (Footwall Cyclic Unit) consists of the FW3 (Footwall 3: norite cycles and alternating pyroxenite-norite-anorthosite subunits, which show interaction of magma with local sedimentary rocks towards the base (parapyroxenite above chromitite interval)); and the UG2CU (UG-2 Cyclic Unit) consists of the UG2HW (UG-2 hanging wall: mineralized feldspathic pyroxenite), the UG-2 (UG-2 analogue: mineralized chromitite interval), and the UG2FW (UG-2 footwall: mineralized pegmatoidal feldspathic harzburgite)

**Fig. 2:**
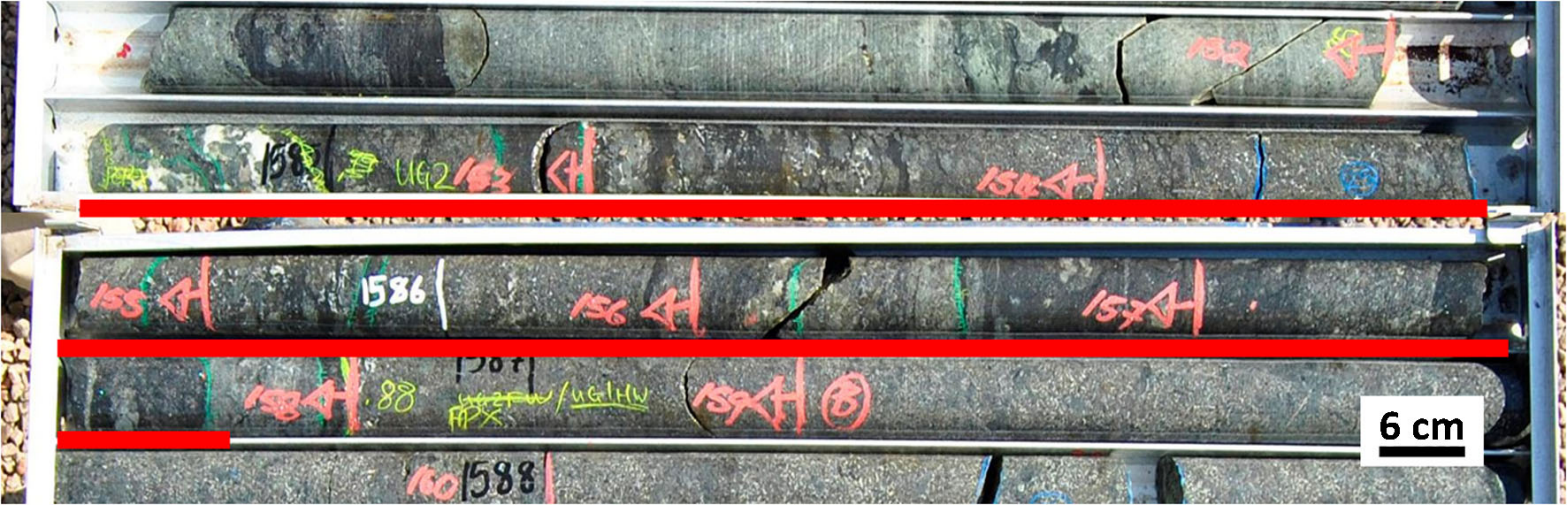
The UG-2E chromitite seam interval in drill-hole UMT366 is indicated by the red underline. The hanging wall consists of feldspathic pyroxenite; dark sections are parapyroxenite (assimilated material). The UG-2E chromitite seam is intersected at a depth of 1584.88 to 1586.77 m (approx. 189-cm-thick); it is comprised of massive to semi-massive chromitite, disseminated chromite, and sections having chromitite stringers and chromitite patches (darker sections are areas with more massive chromitite). The immediate footwall consists of feldspathic pyroxenite

Two sets of samples were used in this study: a reference suite of UG-2 chromitite from the western limb of the BIC and a sample suite of the UG-2E from the northern limb. The reference UG-2 chromitite is the Waterval suite documented in Naldrett et al. (2012); it consists of a 60-cm-thick chromitite seam sampled at 3-cm intervals and mounted in epoxy (for a total of 19 samples). The UG-2E chromitite suite was sampled from drill-core UMT366 (Fig. 1) and consists of a 189-cm-thick interval with variable textures, ranging from massive to semi-massive chromitite, disseminated chromite, and sections having chromitite stringers and chromitite patches (Fig. 2). The UG-2E was intersected in drill-hole UMT366 (Fig. 1), approximately 70 m below the Merensky Cyclic Unit at a depth of 1584.88 to 1586.77 m. The entire interval was sampled from NQ core (4.7 cm diameter) from which a set of 49 polished sections was made. Although the UG-2E in UMT366 has a lower PGE content relative to the UG-2 in the eastern and western limbs, other chromitite intersections from the Turfspruit project have a significant grade up to 6 ppm Pt + Pd + Au + Rh (Grobler et al. 2019).

Samples from both chromitite seams were characterized petrographically under reflected light. Modal proportions were estimated visually and using imaging software ImageJ version 1.51i. Major element contents (Al_2_O_3_, Cr_2_O_3_, MgO, Fe as FeOt) were obtained by electron probe microanalysis (EPMA). Analyses were performed at the Ontario Geological Survey Geoscience Laboratories (GeoLabs) using wavelength dispersive spectrometry (WDS) with a Cameca SX100 EPMA. Major elements (Al, Cr, Mg, Fe) were analyzed with a 2-μm beam diameter at 20 kV and 20 nA. A set of minor and trace elements (Si, Ti, V, Mn, Co, Ni, Zn) were analyzed at 20 kV and 200 nA using a focused beam. Counting time ranged from 20 to 30 s for both on peak and on background positions. Further analyses were performed at the University of Toronto using WDS with a JEOL JXA-8230 probe. All elements were analyzed at 15 kV and 30 nA using a focused beam of 1 μm diameter. GeoLabs (Roberts Victor, chrRV) and University of Toronto (Chrom_PS97) in-house reference mineral standards were used. Calibration for peak positions was performed on diopside (Mg), spinels (Cr, Zn, Al), pure metals (Co, Ti, V), and synthetic metal oxides (Mg, Ti, V, Cr, Mn, Fe, Co, Zn, Al). Matrix corrections were done using the PAP correction procedure (Pouchou and Pichoir 1984) in both cases. Ferric iron contents were calculated from spinel stoichiometry (Droop 1987). To ensure accuracy and reproducibility, multiple samples were reanalyzed in both laboratories. Peak overlap corrections (V Kα and Ti Kβ; Fe Kβ and Co Kα) were performed by the Cameca and JEOL software in each facility. Core and rim analyses showed no significant variations and corroborated that the chromites are compositionally homogeneous.

Trace element contents were determined by LA-ICP-MS at the Harquail School of Earth Sciences using a Resonetics Resolution M50 excimer laser (193 nm) coupled to a Thermo X-Series II quadrupole ICP-MS. A carrier gas of He-N_2_ was used to transport the ablated material from the laser to the ICP-MS. The laser beam used was 90 μm in diameter with an energy density of 4.6 J/cm^2^ and repetition rate of 10 Hz. Ablation time was 40 s of signal preceded by 20 s on background. The masses analyzed correspond to ^24^Mg, ^25^Mg, ^27^Al, ^29^Si, ^31^P, ^34^S, ^45^Sc, ^47^Ti, ^49^Ti, ^51^V, ^52^Cr, ^53^Cr, ^55^Mn, ^59^Co, ^60^Ni, ^63^Cu, ^65^Cu, ^66^Zn, ^71^Ga, ^74^Ge, ^89^Y, ^90^Zr, ^92^Zr, ^93^Nb, ^95^Mo, ^111^Cd, ^118^Sn, ^178^Hf, ^181^Ta, ^182^W, ^195^Pt, and ^208^Pb. Dwell time was 10 μs for all masses. Certain elements in the suite (e.g., S, Cu, Pt, Si) were used mostly to track the presence of inclusions. Calibration was performed on certified reference material NIST SRM 612. The data were processed using Iolite package (on Igor Pro) using the limit of detection methods from Pettke (2008). Standard reference materials included the following: NIST SRM 610; NIST SRM 612; BHVO2G; GOR128; GOR132; GSD; GSC; KL2; and MLB3. Typically, four standards were ablated at the beginning and after every 10 samples in each analytical session. The NIST SRM 612 glass was used as the primary standard for data processing, using the Al content (from EPMA) as the internal standard for calibration. Two compositional maps on grains from the UG-2 reference chromitite (using a 19-μm laser beam) showed no compositional variations from rim to core.

Because ferric iron content estimates from EPMA may not be sufficiently accurate (Evans 2017; Bénard et al. 2018), some samples from the UG-2 and UG-2E were analyzed by Mössbauer spectroscopy at the Swedish Museum of Natural History using a conventional spectrometer system operated in constant acceleration mode. Clean chromite crystals were hand-picked under microscope and ground under acetone in an agate mortar. To avoid other oxide phases, the powdered samples were checked by X-ray diffraction (XRD). Mössbauer absorbers were prepared by grinding 5–13 mg of sample material that was mixed with approximately 75 mg acrylic resin and then pressed into 12-mm-diameter discs under mild heating. Spectra were collected at room temperature using a standard ^57^Co source in a Rh matrix with a nominal activity of 50 mCi. All spectra were acquired over 1024 channels in the velocity range − 4.5 to + 4.5 mm/s and calibrated against an α-Fe foil before folding. The least squares fitting software MossA 1.01f (Prescher et al. 2012) was used to analyze the obtained spectra using different fitting models. Several studies have shown that the recoil-free fractions for Fe^2+^ and Fe^3+^ are unequal (e.g., De Grave and Van Alboom 1991; Eeckhout and De Grave 2003) especially at room temperature. Therefore, the absorption area ratios obtained for the Fe^2+^ and Fe^3+^ doublets were corrected for unequal recoil-free fractions based on the data presented in De Grave and Van Alboom (1991) and the composition of the studied samples, using recoil-free fractions of 0.687 for Fe^2+^ and 0.887 for Fe^3+^ for room temperature measurements.

## Results

### Petrographic characteristics

**Fig. 3:**
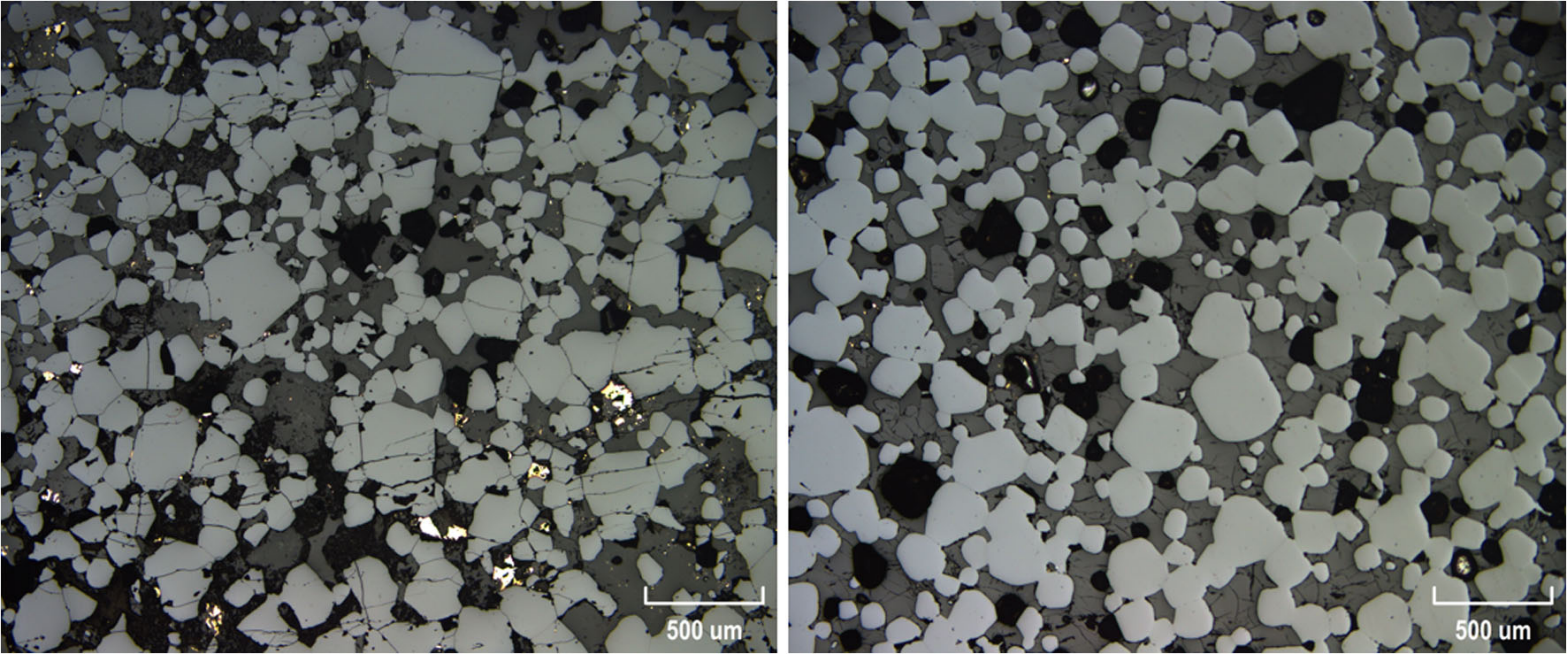
Photomicrographs of UG-2 reference and UG-2E chromites (reflected light). **a** Massive portion of UG-2E chromitite; thin section showing some preferential clustering of larger chromite grains to smaller chromite. Chromite occupies approximately 95% of section (light gray minerals), and sulfides occupy 1% of section (bright anhedral minerals in matrix), whereas silicate minerals take up 4% of section (dark gray minerals making up matrix). Euhedral to subhedral to chromite grains, with grain sizes ranging from 0.04 to 0.7 mm wide. **b** UG-2 chromite grains occupy 92% of section, tiny speckles of sulfide occupy 0.5%, whereas interstitial silicate minerals take up approximately 7.5%. Chromite grain sizes range from 0.01 to 0.25 mm. Lighter gray due to different brightness adjustment

A comparison of representative examples of the massive chromitites is shown in Fig. 3. The UG-2E chromitite has variable chromite content ranging from massive to semi-massive to disseminated chromite (Fig. 2). Only 20% of the interval sampled is massive (> 80% modal chromite; Fig. 3a); some patches contain less than 10% modal chromite. The chromite grain diameter ranges from 0.04 to 1.6 mm and grain habit range from euhedral to subhedral, but occasional anhedral grains are observed. The interstitial spaces are filled mostly by pyroxene, plagioclase, and sulfide minerals. The UG-2 reference chromitite is consistently massive (> 90% modal chromite; Fig. 3b) with euhedral to subhedral chromite grain diameter ranging from 0.001 to 0.6 mm. Like the UG-2E chromite, the interstitial space is filled mostly by pyroxene and plagioclase. In addition to the variations in chromite modal proportions, some other features are different in the UG-2E chromitites compared with those in the UG-2 chromitite. For instance, UG-2E chromite grains are more fractured and often contain secondary material within the fractures (mostly magnetite and serpentine). In contrast, chromite grains from the UG-2 sample are rarely fractured and do not contain any detectable minerals in fractures. Also, sulfides are more abundant in the UG-2E (from ~ 1 to 5%) whereas the UG-2 reference chromitite contains only rare sulfide (≤ 1%). Sulfides are mostly interstitial, but some occur as inclusions in chromite, particularly in the UG-2E samples. The sulfides are typically present as anhedral composite grains of chalcopyrite, pyrrhotite, and pentlandite. Ilmenite exsolutions and rutile are slightly more abundant in the UG-2 chromite than in the UG-2E. Pyroxene is always the dominant silicate mineral in the UG-2E. On average, pyroxene represents 80% of all silicate minerals associated with chromite, with plagioclase representing around 20%. Some thin sections contain olivine (less than 1% but one section had up to 10% modal olivine).

### Mineral chemistry

**Fig. 4:**
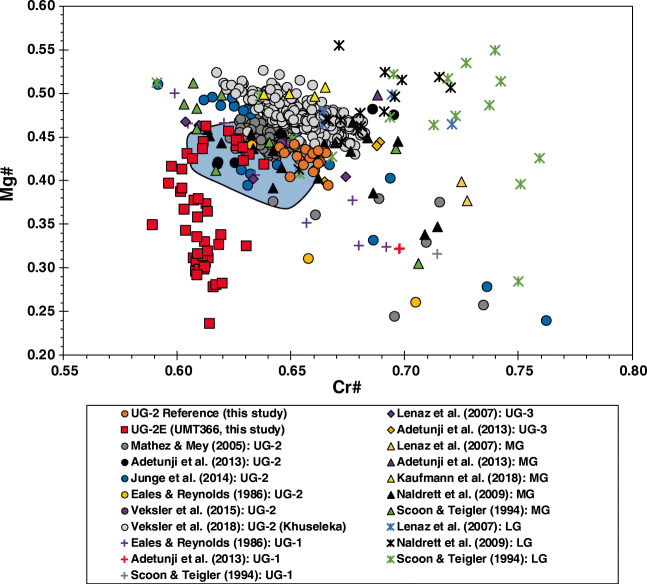
Plot of Mg# (Mg/(Mg + Fe^2+^)) vs. Cr# (Cr# = Cr/(Cr + Al)) for chromites in massive chromitites from the Lower Group (LG), the Middle Group (MG), and Upper Group (UG) of the Bushveld Complex. Note general overlap of UG-2 chromite chemistry from the two limbs, as well as the overlap with the UG-2 reference. Also note the direct overlap of UG-2E chromite data for samples with Mg# ≥ 0.4 and Cr# ≥ 0.62. Chromite from the LG-MG-UG show distinct, but sometimes overlapping chemistry, with MG chromites showing the most overlap with UG and UG-2E chromites

**Fig. 5:**
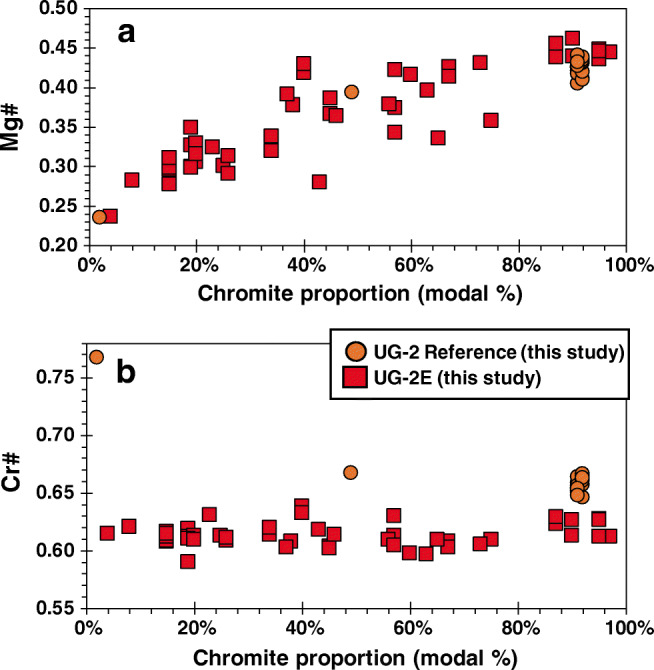
Variations in major cations of chromite in the UG-2 and UG-2E. **a** Variations in Mg# with increasing chromite modal proportions for the UG-2 and UG-2E samples. **b** Variations in Cr# with increasing chromite modal proportions for the UG-2 and UG-2E

**Fig. 6:**
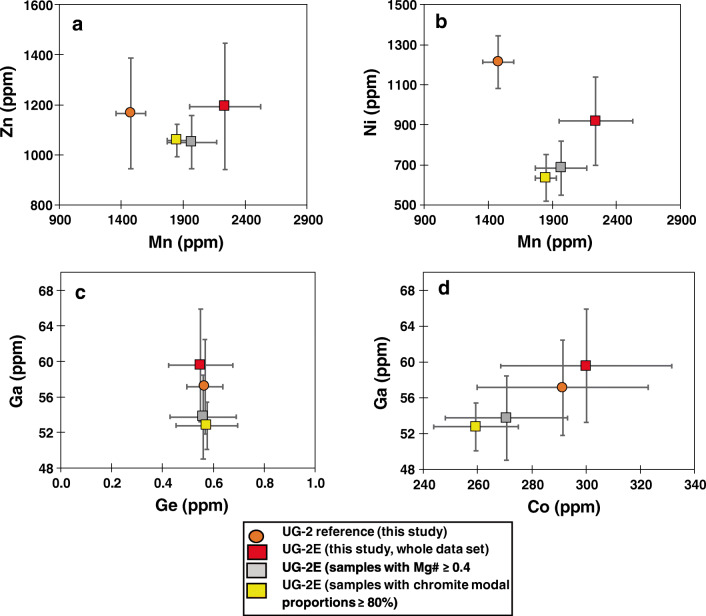
Comparison of minor and trace element LA-ICP-MS data from the UG-2 and UG-2E samples in this study. **a** Zn vs. Mn; **b** Ni vs. Mn; **c** Ga vs. Ge; **d** Ga vs. Co. Minor and trace elements show overlap, especially for trivalent cations. Error bars are 1σ

Major element chemistry of chromite is summarized in ESM Tables 1 and 2 and shown in Figs. 4 and 5. Trace element mineral chemistry is summarized in ESM Tables 3 and 4 and shown in Fig. 6. In general, chromite from the UG-2E has higher TiO_2_, Al_2_O_3_, MnO, FeO_t_, CoO, ZnO, and Fe^3+^/∑Fe than chromite from the UG-2 but lower V_2_O_3_, Cr_2_O_3_, MgO, and NiO, as well as Cr# and Mg# (Fig. 4). The major element composition of the reference UG-2 and UG-2E chromite samples is shown in a Mg# vs. Cr# diagram (Fig. 4) together with chromitite data compiled from the literature for LG, MG, and UG. The results show that the values obtained in this study for the reference UG-2 are consistent with previously published data for the UG-2, which define a narrow cluster centered around Cr# = 0.61 to 0.68 and Mg# = 0.40 to 0.53 with some scattered data defining a trend subparallel to the join between end-member spinel (Cr# = 0; Mg# = 1) and chromite (Cr# = 1; Mg# = 0). The major element composition of the UG-2 chromites is broadly similar, but, in general, eastern limb samples seem to have slightly lower Cr# and Mg# than western limb samples.

In contrast to the UG-2 samples, the UG-2E chromites have a wider range of Mg# but a relatively narrow range of Cr# and only a subset of samples overlap the range defined by UG-2 samples (with Cr# and Mg # roughly around 0.63 and 0.44, respectively) although the overlap is with data documented in the literature and not with the UG-2 samples analyzed in this study.

## Discussion

Using chromite chemistry to attempt a correlation between the UG-2 and the UG-2E is suitable only if the UG-2 can be distinguished from other chromitite layers. Figure 4 shows that, in general, there is a weak trend from higher Cr# and Mg# for the LG chromites towards lower Cr# and Mg# for UG-2 chromites, with some significant overlap between MG and UG chromites (Fig. 4). Naldrett et al. (2012) identified two different trends in Cr# vs. Mg# diagrams and assigned “trend A” to the systematic decrease in Mg# with increasing Cr# and “trend B” to the systematic decrease in Mg# with decreasing Cr#, illustrating the overall changes in composition from LG to UG chromites (Fig. 4). UG-1 and UG-3 chromite data from Eales and Reynolds (1986) and Lenaz et al. (2007), respectively, show overlap with UG-2 data and have a “trend A” that parallels that of UG-2 samples. UG-1 and UG-3 data from the literature are sparse and poorly documented regarding location within the chromitite seam (e.g., margin vs. core).

Regardless of the mechanisms responsible for the different trends, at least three different observations can be extracted from the data distribution in Fig. 4. First, there are systematic compositional differences between the three major chromitite groups (Teigler and Eales 1993). Second, although there is a significant overlap between MG and UG-2 chromite compositions, the MG samples have a larger spread than the UG-2 samples. Third, the UG-2E suite defines a trend that is not consistent with either trend A or trend B defined by Naldrett et al. (2012). Instead, the UG-2E samples define a trend of almost constant Cr# with variable Mg#. A closer inspection of the compiled data (Junge et al. 2014; Mathez and Mey 2005; Veksler et al. 2018) reveals that chromites from the core of the chromitite seams, which are the most massive portion of the chromitites, are almost invariably characterized by Mg# > 0.4, whereas chromite grains at the margins of the chromitite seams have Mg# < 0.4.

To understand the possible reasons for these variations, it is useful to review the concept of trapped liquid shift effect (TLSE). Barnes (1986) introduced the concept to explain how the final composition of cumulate minerals would change from the initial composition because of interaction with evolving intercumulus melt. One key conclusion from Barnes (1986) is that the magnitude of the shift depends mostly on the relative abundance of silicate melt to cumulate crystals and that continuous re-equilibration with intercumulus melt would decrease the Mg# in silicates. Hence, we conclude that chromites at the edges of the chromitite seams (and disseminated chromite) tend to have Mg# < 0.4 mostly because of the TLSE. However, if the modal proportion of the cumulus mineral is high, the effect is small but not negligible, and the composition of the cumulus mineral will be closer to its initial, primitive, composition because there may not be enough interstitial silicate melt to significantly change the composition of the mineral. An important consequence of the TLSE is that comparing chromite mineral chemistry from different chromitite seams is more reliable if samples with similar, and relatively high, chromite modal abundances are used.

It can be argued that contrasts in chromite chemistry can be explained by differences in the relative modal proportion of surrounding minerals, Mg# re-equilibration with pyroxene or olivine, and Cr# re-equilibration by Al exchange with plagioclase. Some studies (Cameron 1975, 1977; Hatton and von Gruenewaldt 1985; Eales and Reynolds 1986; Yudovskaya and Kinnaird 2010; Jones 2013; Junge et al. 2014) argued that systematic changes in disseminated chromite chemistry are controlled by the dominant silicate minerals. However, the compositional changes seem contradictory, and the compositional changes in chromite chemistry do not seem systematic enough to distinguish among chromites surrounded by any specific silicate mineral (Veksler et al. 2015, 2018). For the UG-2E samples in this study, the relative pyroxene to plagioclase proportion was consistently around 80:20, meaning that variations in Mg#, Cr#, or Fe^3+^/ΣFe are not influenced by the relative proportion of surrounding silicates.

Figure 5 shows the variations in Cr# and Mg# against chromite modal proportions. The UG-2 reference chromitite seam is predominantly massive (≥ 90 modal chromite) and Mg# and Cr# (Fig. 5a, b, respectively) show little change except for two samples with 49% chromite and 2% chromite (UG-2-1-21 and UG-2-1-2, respectively), with UG-2-1-2 being mostly disseminated chromite in a thin silicate stringer near the top chromitite margin. The change in Mg# with decreasing chromite modal proportion is evident for the UG-2E chromitite. In this case, the chromitite seam ranges from massive to semi-massive to disseminated (Fig. 2) with a wide range in chromite modal proportions (from < 10 up to > 90%) and a wide range in Mg # (from 0.23 to 0.45). It is relevant to note that the Mg# of UG-2E samples with more than 80% chromite overlap the Mg# of the UG-2 reference samples (Fig. 5a). In contrast, Fig. 5b shows that although variations in chromite modal proportion seem to affect the Cr# of the UG-2 reference suite (up to 0.77 for a sample with only disseminated chromite), it does not seem to affect the Cr# of the UG-2E samples, which remain at around 0.63 regardless of the chromite modal abundance. If filtered by chromite modal proportion, the UG-2E samples with more than 80% chromite have Cr# slightly lower than the UG-2 reference. However, when compared with compiled UG-2 data (Fig. 4), the Cr# and Mg# of the UG-2E matches the field of the UG-2, especially the field defined by samples from the Nkwe in the eastern limb. A subset of UG-2E samples, with Cr# > 0.62 and Mg# > 0.40, also shows the “trend A” of decreasing Mg# with increasing Cr# identified in other Bushveld samples (Naldrett et al. 2012), which is consistent with the interpretation of the UG-2E as being correlative to the UG-2 in the northern limb.

In an attempt to provide other ways to compare the UG-2 and the UG-2E, trace elements by LA-ICP-MS were obtained. Some highlights of the results are shown in Fig. 6. One advantage of LA-ICP-MS is that it provides better data quality for minor and trace elements such as Mn, Co, Ni, V, and Zn than EPMA. One difficulty in evaluating trace element content is the lack of data from the LG and MG suites to provide context for comparison. UG-2 and UG-2E chromites have the same Zn content, within uncertainty, but slightly different Mn content (Fig. 6a) even when chromite with Mg# < 0.4 is removed from the dataset. In contrast, there are significant differences in Ni content (Fig. 6b) with the UG-2 samples having roughly twice the Ni content in chromite (1210 ± 130 ppm) than chromite from the most massive UG-2E samples analyzed. There are similar contents in Ga and Ge (Fig. 6c) and significant overlaps in Co contents (Fig. 6d).

**Fig. 7:**
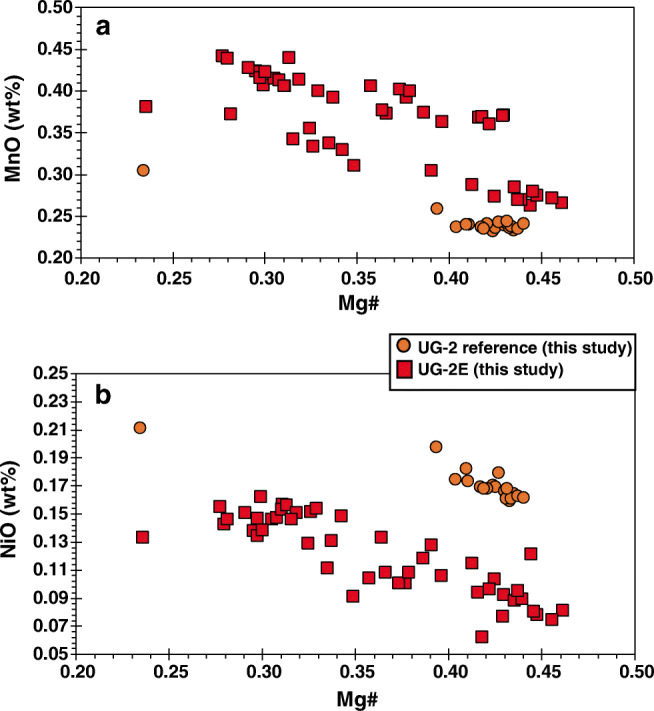
Variations in minor element contents with changing modal chromite abundance. **a** Variations in MnO with Mg# for the UG-2 and UG-2E. **b** Variations in NiO with Mg# for the UG-2 and UG-2E. In general, the lower the modal chromite abundance, the higher the minor element contents of NiO and MnO for both the UG-2 and UG-2E

**Fig. 8:**
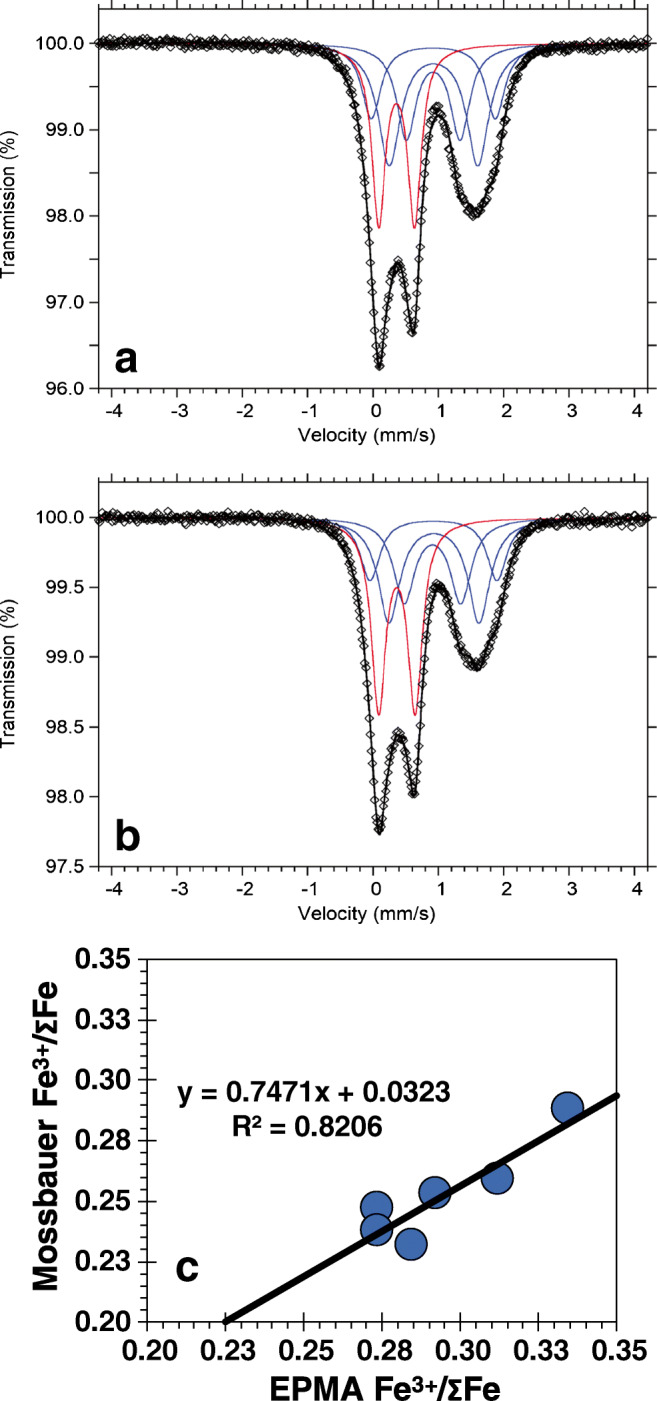
Fe-Mössbauer spectra. **a** Sample UG-2E-48. **b** Sample UG-2E-29. All spectra were obtained at room temperature. Fitted absorption doublets assigned to Fe^2+^ and Fe^3+^ are indicated in blue and red, respectively. Diamonds denote measured spectrum, and black curve represents summed fitted spectra. **c** Comparison of Fe^3+^/∑Fe UG-2 reference and UG-2E sample data from EPMA and Mössbauer spectroscopy

Although massive sections of the UG-2E match the composition and trend of the UG-2, the rest of the UG-2E samples define a trend of roughly constant Cr# and variable Mg# that is inconsistent with trends A and B identified previously for the BIC chromitites (Naldrett et al. 2012). A possible explanation for this is that, despite the TLSE, the chromitites crystallized in a closed system in the eastern and western limbs with no contamination from local footwall rocks, whereas in the northern limb, the magmas from which the UG-2E formed likely interacted, assimilated, and were contaminated by footwall rocks from the Transvaal Supergroup. The match in composition between UG-2 and UG-2E for massive chromitite sections indicates that at least some chromite crystallized before significant assimilation. Porosity and permeability were limited enough so that the effects of the TLSE were no different than in the rest of the BIC. In contrast, the less massive parts of the UG-2E interacted with magma that was likely contaminated by footwall assimilation, hence creating trends in Cr# vs. Mg# diagrams that are not consistent with trends A and B. Variations in minor elements with changes in Mg# may help assess this possibility. Figure 8 shows variations in MnO content against Mg# (Fig. 7a) and variations in NiO against Mg# (Fig. 7b). Both figures show negative trends of increasing MnO and NiO contents with decreasing Mg# for the UG-2E suite, and a similar trend in NiO content, but no change in MnO content, for the UG-2 suite. The higher MnO contents in UG-2E samples may indicate addition of MnO during footwall assimilation. The reason for the contrast in NiO content is not clear. Both suites of samples show roughly the same trends, but the NiO content is higher in UG-2 samples compared with that in UG-2E samples with Mg# > 0.4, from the most massive sections of the UG-2E. One possible explanation is that the Ni content in chromite is significantly affected by coexistence with sulfides, mainly pentlandite. If the UG-2E suite coexisted with a larger proportion of sulfides, it would be expected that Ni would be preferentially partitioned into the sulfide phases leaving chromite with lower Ni content than similar chromite that coexisted with lesser amounts of sulfides. This explanation is consistent with the relative abundances of sulfides observed, because the modal abundance of interstitial sulfides is higher in the UG-2E than the in the UG-2.

Additional understanding of the processes affecting the UG-2E can be gained by investigating the ferric iron content in chromite. Figure 8 shows the Mössbauer spectra obtained for samples UG-2E-48 and UG-2E-29 (Fig. 8a, b, respectively), with Fig. 8a showing the most reduced samples in the suite and Fig. 8b the most oxidized. The spectra are similar albeit broader than those previously reported for UG-2 samples from the eastern limb (Adetunji et al. 2013). The Fe^3+^/ΣFe ratios range from 0.23 to 0.29 (ESM Tables 1 and 2) and were used to assess systematic variations in the Fe^3+^/ΣFe estimated from EPMA data (Fig. 8c). Only six samples were analyzed by Mössbauer spectroscopy but the comparison indicates that although Fe^3+^/ΣFe estimates from EPMA data are about 14% higher than what is estimated from Mössbauer spectroscopy, the positive linear correlation (*r*^2^ = 0.82) allows for correction of EMPA estimates using the equation derived from the linear regression (Fig. 8c).

**Fig. 9:**
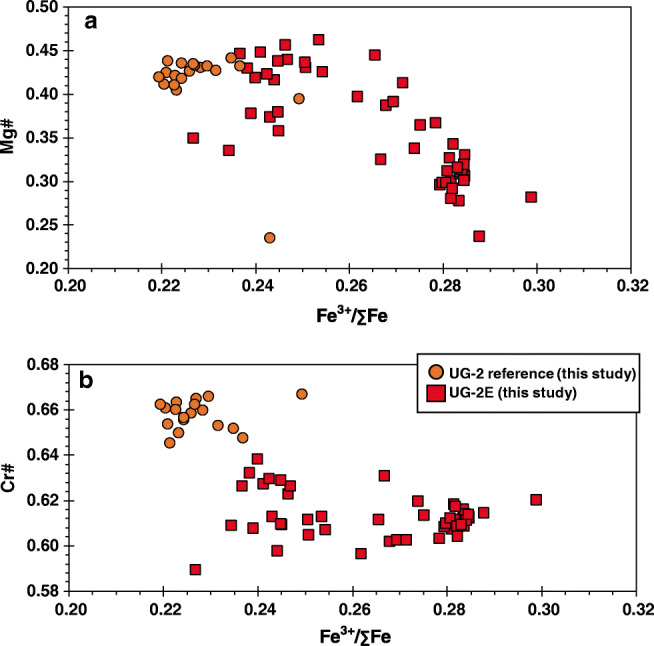
Variations in Fe ratios with changing modal chromite abundance. **a** Variations in Mg# with Fe^3+^/∑Fe for the UG-2 and UG-2E. **b** Variations in Cr# with Fe^3+^/∑Fe for the UG-2 and UG-2E. UG-2 reference chromites have lower Fe^3+^/∑Fe compared with UG-2E chromites. Fe^3+^/∑Fe derived from correction of EPMA data by Mössbauer spectroscopy

The variations in Mg# against Fe^3+^/ΣFe estimates (Fig. 9a) show that the UG-2 samples are relatively reduced compared with the UG-2E suite and that UG-2E samples with lowest Mg# correspond to samples with the highest Fe^3+^/ΣFe. In contrast, there are no systematic variations in Cr# as a function of Fe^3+^/ΣFe (Fig. 9b). The wider spread in Fe^3+^/ΣFe for the UG-2E suite compared with that for the UG-2 is interpreted as good evidence that the UG-2E magmas assimilated variable amounts of crustal material, making the magma more oxidized than the UG-2 magma. However, chemical equilibrium was not reached as this would have homogenized the composition and shifted the Fe^3+^/ΣFe to a much narrower range than what is recorded in the samples. This could happen if the magmas cooled relatively fast soon after assimilation at the level of emplacement.

### Implications for correlation of northern limb lithologies and petrogenetic processes

Our compilation shows a remarkable consistency in major element composition (Mg#, Cr#) of the UG-2 seam between the eastern and western limbs, which are more than 300 km apart (Eales and Cawthorn 1996). Thus, if the UG-2 were to occur in the northern limb, it is reasonable to assume that its composition would be expected to be consistent with the UG-2 elsewhere in the BIC. However, the opposite is not necessarily true, as chromite matching UG-2 composition may not be UG-2. This is mostly evident by the overlap in composition from the MG group (e.g. Kaufmann et al. 2018). Conversely, a poor match between UG-2E and UG-2 chromites (of comparable modal proportions) could be used as an argument against the UG-2E being the equivalent of the UG-2. We established that, in high chromite modal proportions (> 80%), the major element content of chromite from the UG-2E matches the major element content of chromite from UG-2. Other lines of evidence support the interpretation of the UG-2E as the equivalent of the UG-2. First, with the exception of the UG-3, which has been documented in a few localities in the northern part of the eastern limb, the UG-2 is the uppermost chromitite seam in the UCZ, occurring tens of meters below the Merensky Reef. In the northern limb (at Turfspruit), the UG-2E matches that relationship with the main mineralization in the Platreef, which has been interpreted as being the Merensky Reef equivalent (Grobler et al. 2019). Also, Mayer et al. (submitted) document systematic ^87^Sr/^86^Sr_i_ variations across the mineralized section of the Platreef that match similar variations in the main limbs of the BIC (Seabrook et al. 2005). Other lines of evidence have been used to propose that the UCZ rocks in the northern limb formed from a different magma than the UCZ rocks in the eastern and western limbs. For example, McDonald et al. (2005) documented significant differences in the Mg# of orthopyroxene and olivine from the Platreef compared with Merensky Reef samples and used this, along with Pt/Pd ratios, to conclude that the UCZ in the northern limb could not be correlated with the UCZ in the rest of the BIC. Although it remains a possibility that the UCZ in the northern limb was formed by a distinct type of magma, the combined lines of evidence that are available now are more consistent with the interpretation of the UCZ in the northern limb as being not only the equivalent of the UCZ in the rest of the BIC but also the expression of the same magmas, with the main differences being due mostly as an effect of variable degrees of assimilation of crustal material at, or near, the level of emplacement. The assimilated crustal material in this locality would consist of pelites, shales, dolomites, and banded iron formation of the Duitschland Formation of the Transvaal Supergroup; some evidence of this assimilation is observed in drill core (Fig. 2; immediate hanging wall of UG-2E). The Mg# and Cr# of the UG-2E chromites are likely to have been modified by incorporation of either Fe (both as Fe^2+^ and Fe^3+^), Mg, and Al from the Transvaal sedimentary rocks during chromite crystallization. The assimilation of crustal material most likely coincides with lower chromite abundances as a result of the cessation of chromite crystallization in between several periods of magma recharge. The portions with lower chromite abundances would then re-equilibrate with surrounding silicate minerals. Therefore, the decrease in Mg# with relatively constant Cr# seems to be associated with contamination, defining a trend that is distinct from trends A and B shown by Naldrett et al. (2009).

## Conclusions

The results demonstrate that there is overlap of the chromite Cr# and Mg# between samples of the UG-2 and UG-2E when samples with > 80% modal chromite proportions are compared. This and other evidences, such as stratigraphic position of the UG-2E below the main PGE mineralization in the Platreef, are consistent with the interpretation that the UG-2E crystallized from a magma of the same composition as the UG-2, and therefore, the UG-2E is likely the UG-2.

Geochemical differences (major, minor, and trace elements) between the UG-2 and the less massive portions of the UG-2E are most likely due to assimilation of metasedimentary footwall rocks together with re-equilibration with variable amounts of interstitial silicate melt. This was marked mostly by a significant decrease in the Mg#.

Regardless of whether the trapped-liquid shift effect has affected the samples, major element chemistry (Cr# vs. Mg#) is a useful correlation tool in chromite samples with more than 80% chromite.

## Electronic supplementary material

ESM 1 (XLSX 68 kb)
